# Effect of Cold Chain Logistic Interruptions on Lipid Oxidation and Volatile Organic Compounds of Salmon (*Salmo salar*) and Their Correlations With Water Dynamics

**DOI:** 10.3389/fnut.2020.00155

**Published:** 2020-09-09

**Authors:** Ying-Jie Yu, Sheng-Ping Yang, Ting Lin, Yun-Fang Qian, Jing Xie, Changli Hu

**Affiliations:** ^1^College of Food Science and Technology, Shanghai Ocean University, Shanghai, China; ^2^Shanghai Engineering Research Center of Aquatic Product Processing and Preservation, Shanghai, China; ^3^National Experimental Teaching Demonstration Center for Food Science and Engineering (Shanghai Ocean University), Shanghai, China; ^4^Shanghai Professional Technology Service Platform on Cold Chain Equipment Performance and Energy Saving Evaluation, Shanghai, China; ^5^Nanjing Weigang Dairy Co., Ltd, Nanjing, China

**Keywords:** salmon filets, cold storage, temperature fluctuations, low-field nuclear magnetic resonance (LF-NMR), fatty acid, volatile organic compounds (VOCs)

## Abstract

Lipid oxidation and water migration are important factors in the quality changes of aquatic products. This study investigated the relationship between water migration and lipid oxidation in salmon filets under four different storage conditions (control: 0°C; T1: 4°C; T2 and T3: two temperature fluctuation groups) by detecting thiobarbituric acid–reactive substances, changes of fatty acids, and volatile organic compounds (VOCs), and other quality indicators including redness, microorganism, total volatile basic nitrogen (TVB-N), and water-holding capacity (WHC) were also measured. The results of low-field nuclear magnetic resonance (LF-NMR) showed that more trapped water (T_22_) turned to form free water (T_23_) in groups suffering temperature fluctuations. A more significant decrease in fatty acids was found in T2 and T1 groups, especially oleic acid (C18:1n9c), linoleic acid (C18:2n6c), and palmitic acid (C16:0). The VOCs with off-flavors (1-penten-3-ol, 2-penten-1-ol, (Z)-, 1-octen-3-ol, hexanal) in the groups suffered from simulated cold chain interruptions increased faster than the other two groups during storage. T_22_ was negatively correlated (*p* < 0.05) with stearic acid (C18:0), 1-penten-3-ol, hexanal, and nonanal, whereas T_23_ was positively correlated with 1-penten-3-ol, hexanal, and heptanal. Therefore, the temperature fluctuation accelerated the loss of polyunsaturated fatty acids and the increase of unpleasant odors related to water migration.

## Introduction

Temperature is considered to be the most important environmental factor that inhibits the microbiological growth and thus influences the shelf life of fresh fish (1). However, it is very difficult to keep the temperature constant during the actual cold chain transportation, especially when loading and unloading. In recent years, more and more scientists have paid attention to the effect of temperature fluctuation on the quality of aquatic products. Several storage trials at fluctuating temperature conditions on quality changes of fishery products have been conducted in recent years (2–4).

Salmon (*Salmon salar*) belongs to the family Salmonidae and is a high quality of considerable nutritional and economic importance because of its high content of polyunsaturated fatty acids (PUFAs), including eicosapentaenoic acid (EPA) and docosahexaenoic acid (DHA) (5, 6). However, salmon is highly perishable during transportation and storage, which are contributed not only by the growth of microorganisms and protein degradation, but also by lipid oxidation (7). Lipid oxidation in seafood may result in the production of volatile oxidation products, which will affect its odor, flavor, appearance, texture, and even the nutrients and the shelf life (8). Our previous study also found that salmon was rich in lipid; lipid oxidation not only affected the odor, but also affected the color appearance (9). Lipid oxidation in seafood is usually triggered by enzymes such as lipase, lipoxygenase, cycloxygenase, etc., which needs water as a medium. Unfortunately, water is the most abundant component in tissues of seafood. Although most water is trapped in muscle in fresh seafood, it may migrate because of the degradation of the cell membrane and myofibrillar proteins, leading to the spread of soluble enzymes (10). It is hypothesized that water migration may be correlated with the lipid oxidation and production of volatile organic compounds (VOCs).

Therefore, this article mainly studies the changes of water migration, lipid oxidation, and VOCs caused by temperature fluctuations in salmon during simulated cold chain logistics, as well as the growth of microorganisms, and their correlations were analyzed, so as to demonstrate the relationship between water migration, lipid oxidation, and off-odors.

## Materials and Methods

### Sample Preparation

**Figure 1:**
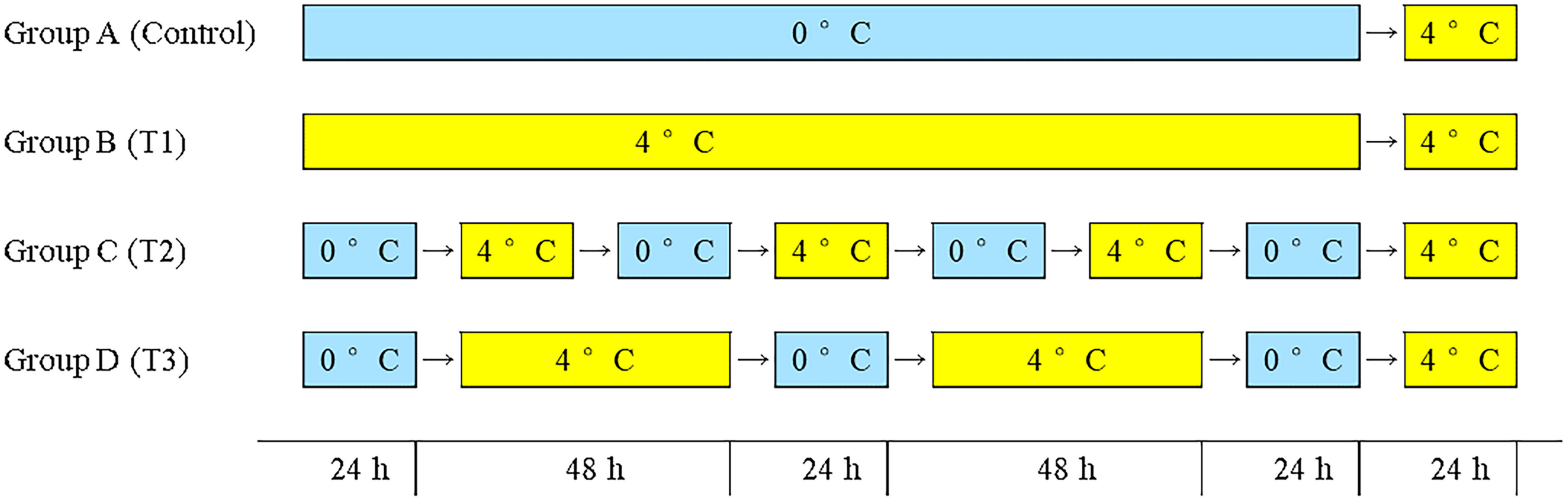
Group A: 0°C constant (control group), group B: 4°C constant (T1 group), group C: seven shifts for 24 h from 0 to 4°C (T2 group), group D: five shifts for 0°C 24 h and 4°C 48 h (T3 group).

The fish in this study were fresh farmed Atlantic salmon (each gutted weighed about 5 kg) were purchased from an aquatic products market (Pudong District, Shanghai, China). The salmon samples were gutted after being caught in the local farms (brand: Super Salmon) and transported immediately in a closed box with ice to Shanghai by air within 3 days. On arrival, all the samples were transferred immediately to the laboratory. After washing with distilled water, the fish was cut into pieces of about 200 g and put them in polyethylene bags (Fanren Packaging Co., Ltd., Foshan, China). The samples were randomly divided into four groups and stored under four simulated logistics conditions for 8 days as shown in Figure 1. Sampling is performed daily. The sampling was carried out every day.

### Sensory Evaluation

Ten persons from the laboratory staff were asked to assess the sensory evaluation of salmon. The total scores of five attributes include color, odor, tissue morphology, elasticity, and mucus, and the quality of the samples based on 5-point hedonic scales in descending order from 5 to 1 (9). The average of the total score of the five attributes is considered the final result, and a total score of lower than two is considered an unacceptable threshold.

### Determination of Redness Value

The redness value (a^*^) of salmon was recorded by a portable colorimeter (CR-400; Konica, Tokyo, Japan). Each batch was conducted in triplicate.

### Microbiological Analyses

Quantitative microbiological analysis was determined according to the method of Xie et al. (11). At each sampling, salmon filet samples (25 g) were homogenized with 225 mL of sterilized saline water (0.85 g/100 mL). One milliliter of these 10-fold dilutions by sterilized saline water was mixed with 15–20 mL of iron agar (no. HB8735; Qingdao Hope Biol-Technology Co., Ltd., Qingdao, PR China). Total mesophilic bacterial counts and psychrotrophic bacterial counts were determined by counting the total colonies on the plate after incubating at 30°C for 48 h and at 4°C for 10 days, respectively. For H_2_S-producing bacteria, the black colonies were counted after 72 h of incubation at 25°C. Each dilution was performed in three parallels.

### Determination of Total Volatile Basic Nitrogen and Thiobarbituric Acid–Reactive Substances

The total volatile basic nitrogen (TVB-N) value was determined according to the method of FOSS (12) by using an Automatic Kjeldahl Apparatus (Kjeltec Analyzer Unit; Foss Tecator AB, Hoganas, Sweden).

The thiobarbituric acid–reactive substances (TBARS) were determined according to the method of Ibrahim Sallam (5) with minor modifications. The fish samples (5 g) were homogenized with 25 mL of 20% (wt/vol) trichloroacetic acid (Sinopharm Chemical Reagent Co., Ltd., Shanghai, PR China), centrifuged, and incubated, and the absorbance was measured at 532 nm. The TBARS value was expressed as mg malondialdehyde equivalents per 100 g of samples (mg/100 g).

### Determination of Water-Holding Capacity

The fish samples (M1) (3 g) were weighed accurately, and the fish samples (M2) were weighed after centrifugation at 3,000 r/min for 10 min. The formula for calculating water-holding capacity (WHC) (%) was as follows:

$$\text{WHC}\%=(\text{1}-\frac{\text{M}1-\text{M}2}{\text{M1}})\times 100\%.$$

### Determination of Transverse Relaxation Time (T_2_) of Low-Field Nuclear Magnetic Resonance and Proton Magnetic Resonance Imaging

T_2_ relaxation measurements were performed using a low-field nuclear magnetic resonance (LF-NMR) analyzer minispec PQ 001 (Niumag, Ltd., Shanghai, China). The fish sample (2 × 2 × 2 cm) was put into the detecting tube (70-mm diameter). Using the Carr-Purcell-Meiboom-Gill sequence to set the T_2_ measurement parameters (9), the analysis software (NMI20-030H-1 NMR analyzer: Suzhou Niumag Analytical Instrument Co., Suzhou, China) was used to iteratively invert the collected signals to obtain the transverse relaxation time T_2_ spectrum. Similarly, the proton density distribution of the sample is determined by magnetic resonance imaging (MRI) after setting the parameters. The gray-scale map of proton intensity was altered to be pseudocolor images by MATLAB software (MathWorks Inc., Natick, MA, USA).

### Fatty Acid Profile

The total lipids of salmon were extracted by adding 10 g of flesh sample with chloroform/methanol (2:1, vol/vol) and then condensed using rotary evaporation according to the method of Folch et al. (13). The extracted sample (0.1 g) in a 100-mL round-bottomed flask and methylate the fatty acids by the boron trifluoride-methanol method according to the method of Zhang et al. (14) and analyze it by gas chromatography (GC) after methyl esterification. Qualitative analysis was performed according to the comparative retention time of fatty acid methyl ester mixed standards (Sigma–Aldrich Co. LLC, USA) and samples, and quantitative analysis was performed by the internal standard method. GC parameters were set according to the method of Merlo et al. (15).

### VOC Determination by Headspace Solid Phase Microextraction Coupled With GC/Mass Spectrometry Analysis

The VOC analysis was measured according to the method of Parlapani et al. (16) with some modifications. Three grams of fish meat was weighed and added it to 20-mL headspace vials and equilibrate at 50°C for 15 min. Then the SPME fiber (DVB/CAR/PDMS 50/30 μm) was exposed to the headspace for additional 40 min. The fiber with VOCs was inserted into the injection port of the GC at 250°C for 5 min.

GC/mass spectrometry (MS) analysis was performed on an Agilent 7890A gas chromatograph coupled to an Agilent 5977A mass spectrometer (Agilent Technologies Co. Ltd., CA, USA). A capillary HP-5MS column (30 m × 0.25 mm × 0.25 μm) was used, and the GC oven temperature initially was maintained at 40°C for 3 min and then was raised to 100°C with a program of 3°C/min, and increase of 5°C/min to a final temperature of 230°C and kept for 5 min. The carrier gas (helium) flow rate was 0.8 mL/min, the analytical temperature was 250°C, and the injection was performed in splitless mode. The temperature of transfer lines was maintained at 280°C, the temperature of MS source and quadrupole was set at 230 and 150°C, respectively. The scan range from 30 to 550 m/z and ionization energy was 70 eV. The VOCs were identified by comparing their retention times with reference compounds or by comparing their mass spectra with those stored in the National Institute of Standards and Technology 11 spectral database. The amount of VOCs was expressed as the peak area of the deconvoluted component multiplied by any unit of 10^−6^.

### Statistical Analysis

At least three measurements of all experiments were performed. The data were performed in Excel 2010 software (Microsoft Corp., Redmond, WA, USA), and analysis of variance and mean comparison were carried out by using SPSS 20 (SPSS version 20.0; SPSS Inc., Chicago, IL, USA); the significant difference was set at *p* < 0.05, and the figures were performed using the Origin2018 software (OriginLab, USA).

## Results and Discussion

### Sensory Evaluation

**Figure 2:**
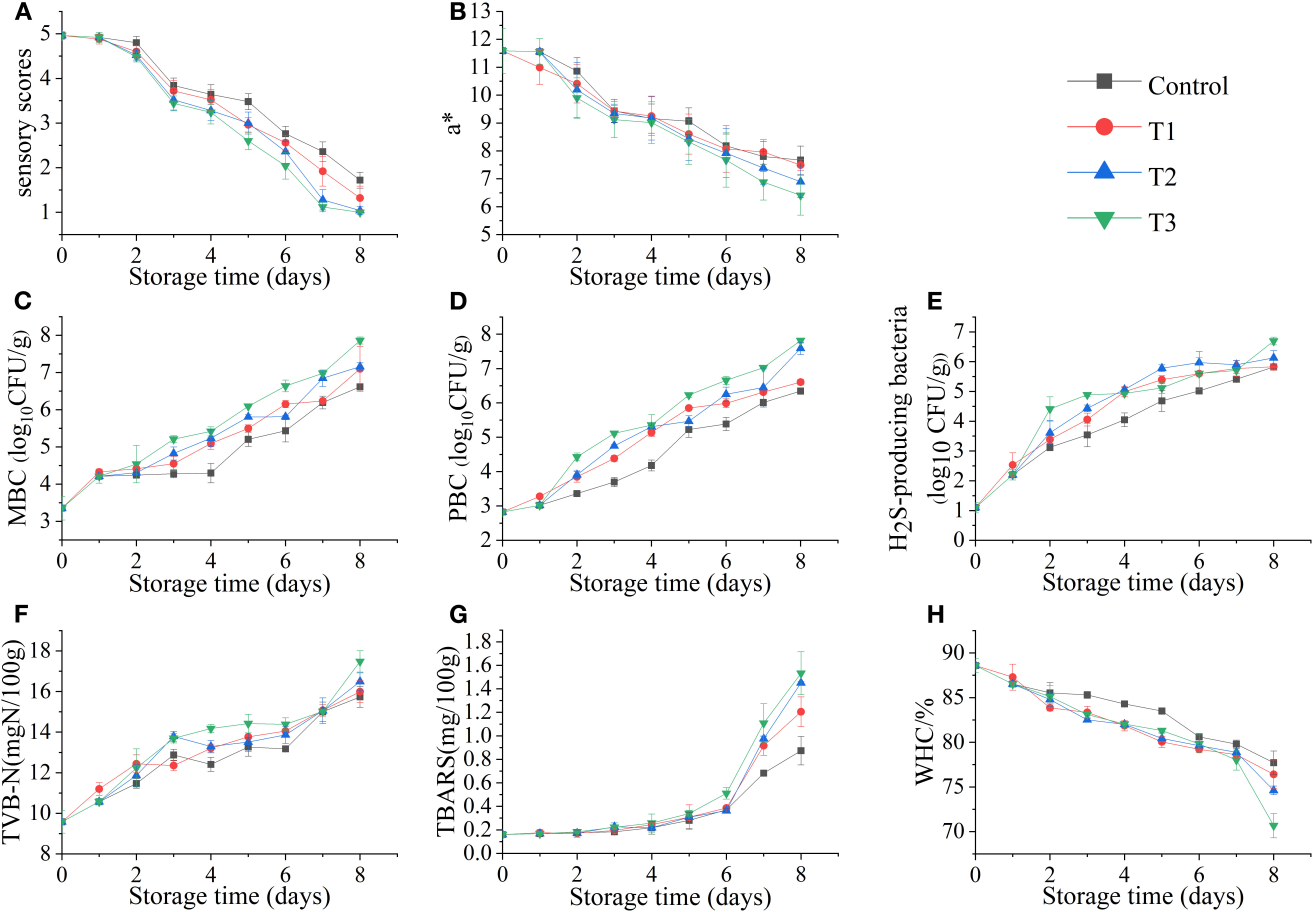
Sensory score **(A)**, redness value (a*) **(B)**, total mesophilic bacteria count (MBC) **(C)**, total psychrotrophic bacteria count (PBC) **(D)**, hydrogen sulfide–producing bacteria count **(E)**, total volatile basic nitrogen (TVB-N) values **(F)**, thiobarbituric acid–reactive substances (TBARS) values **(G)**, and water-holding capacity (WHC) **(H)** of salmon filets under four different storage conditions.

As shown in Figure 2A, the sensory scores of the four groups of fish samples decreased during storage, but the degradation rate of the T3 was significantly faster. On the third day, T3 began to produce mucus, but no off-odor was produced in all four groups. On the 6th day, the mucus in all groups increased significantly, whereas the elasticity decreased, and the color was darker than before; the three groups (control, T1, T2) started to exhibit a slight ammonia odor, but the unacceptable rancid odor began to appear in T3, which led to a significant decrease in sensory scores. On the 8th day, the sensory scores of all groups reached an unacceptable threshold. This phenomenon was similar to the research of Wang et al. (9). The sensory score of salmon at 0°C was significantly higher than the group that suffered temperature fluctuations, and the quality of salmon filets was rapidly decreased because of the temperature fluctuation, and the score was also significantly lower than that of the control group.

### Determination of Redness Value (a^*^)

The fresh salmon filets exhibit bright red in color due to the high astaxanthin content. The change in redness value (a^*^) of salmon filets is shown in Figure 2B. The color of the four groups of fish samples changed significantly with the increase of time. Among them, the colors of T2 and T3 became significantly darker; especially the muscle of T3 was not bright. The redness value decreased with the increasing time, which might be due to the oxidation of astaxanthin (17). The color of salmon filets during storage is related to the astaxanthin state (18). And the decrease in the redness value was also one of the reasons for the decrease in sensory scores.

### Microbiological Analyses

The changes in total mesophilic bacteria count (MBC), total psychrotrophic bacteria count (PBC), and H_2_S-producing bacteria count in salmon filets stored at different storage conditions are shown in Figures 2C–E. In this study, the initial number of MBC was low [<4 log_10_ CFU/g] before the salmon filets were subjected to different treatments, indicating that the fish filets were fresh. It is shown that the MBC count of the control group was significantly lower than that of the other three groups, whereas the number of the T3 group was significantly higher, and the growth rates and numbers of the T1 and T2 groups were closer. The T3 group first reached the microbial limit [ ≤ 7 log_10_ CFU/g, ICMSF (19)] during storage. On the 8th day, the T3 group had reached 7.9 log_10_ CFU/g, and the T1 and T2 groups reached 7.1 and 7.2 log_10_ CFU/g, respectively, whereas the control group reached 6.6 log_10_ CFU/g, which did not exceed the microbial limit. The rapid reproduction of microorganisms should be caused by temperature fluctuations (20).

The psychrotrophic microorganisms are one of the main microorganisms in fishery products during refrigeration (21). It can be seen from Figure 2D that the growth trend of PBC is similar to that of MBC. The T3 group was significantly higher than the other three groups. Similarly, on the 8th day, the PBC of T3 was the highest, reaching 7.8 log_10_ CFU/g, followed by the T2 group also reaching 7.6 log_10_ CFU/g, whereas the control and T1 groups reached only 6.3 and 6.6 log_10_ CFU/g, respectively. The results confirmed that temperature fluctuations would accelerate bacterial growth in seafood.

Most H_2_S-producing bacteria found in fishery products are gram-negative bacteria, such as *Shewanella* spp., *Serratia* spp., *Aeromonas* spp., etc. They can produce trimethylamine (TMA) and H_2_S and amino acid decarboxylation and proteolytic activity (22). The counts of H_2_S-producing bacteria in salmon filets under four storage conditions also increased with storage time, reaching the highest value on the 8th day. The T2 and T3 groups were 6.1 and 6.7 log_10_ CFU/g, respectively, whereas the control and T1 groups were only 5.8 log_10_ CFU/g.

### Determination of TVB-N and TBARS

The TVB-N value is used as one of the important indicators to evaluate the freshness of fishery products (23). As shown in Figure 2F, the TVB-N value of salmon increased during four different storages. The TVB-N value of the T3 group was significantly higher than the other three groups, reaching 17.48 mg N/100 g at the end of storage, followed by the T2, T1, and the control groups in descending order. The increase of TVB-N is mainly related to the activities of spoilage microorganisms and endogenous enzymes in fish (24). The decomposition of protein and some non-protein nitrogen compounds in salmon will become basic nitrogen-containing substances, including ammonia, monoethyl amine, dimethylamine, and TMA, which are also contributing to the odor changes (25).

The TBARS value is one of the important indexes to evaluate the degree of lipid oxidation (26). Because salmon is rich in fat and PUFAs, the lipid oxidation may play an important role in deterioration by producing unpleasant odors and yellowish color (7). As shown in Figure 2G, the TBARS value of salmon increased slowly in the first 6 days, but rapidly in the following 2 days, indicating that lipid oxidation was accelerated at this time. After 8 days of storage, the TBARS values of T2 and T3 groups reached 1.45 and 1.53 mg/100 g, respectively, and the control and T1 groups reached 0.87 and 1.20 mg/100 g, respectively. The TBARS values of the temperature fluctuation group were significantly higher than that of the control group, indicating the temperature fluctuation accelerated the lipid oxidation of salmon.

### Determination of WHC

The WHC of salmon filets decreased with storage time under different storage conditions (Figure 2H). The WHC of fresh salmon was 88.59%, and the WHC of four storage conditions decreased with the increasing time, especially at the end of storage. The WHC of the T3 group decreased to 70.67% on the 8th day, and those of the control, T1, and T2 groups were 77.71, 76.41, and 74.61%, respectively. The oxidation and decomposition of proteins could be the main cause of this phenomenon (27).

### Transverse Relaxation Time (T_2_) of LF-NMR

**Figure 3:**
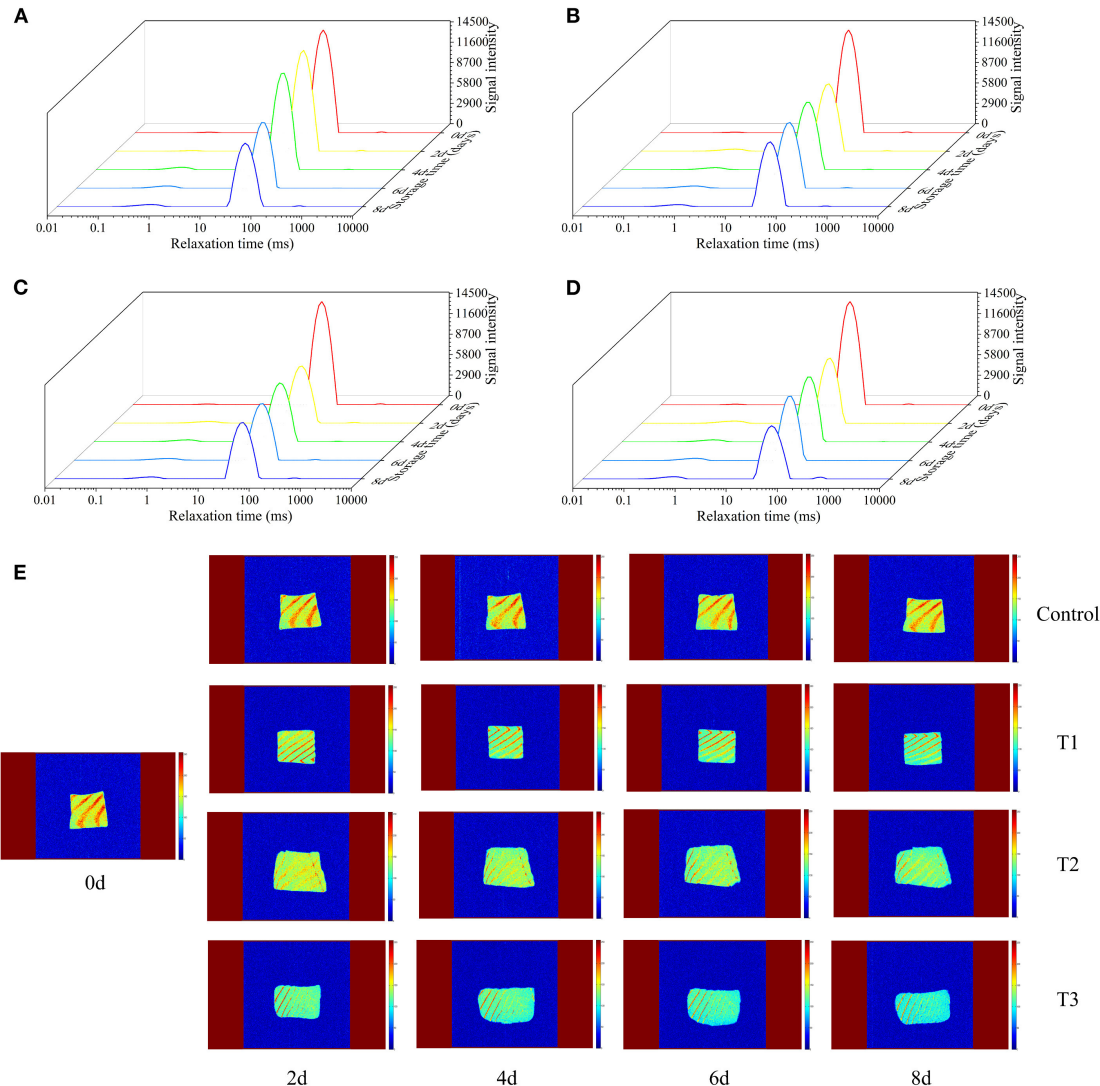
Transverse relaxation time (T_2_) of salmon filets under four different storage conditions **(A–D)** and magnetic resonance imaging (MRI) of salmon filets under four different storage conditions **(E)**.

The transverse relaxation time (T_2_) of the LF-NMR technique can determine the changes in water distribution and water migration of salmon filets under different storage conditions. The original contributions presented in the study are publicly available. These data can be found at https://doi.org/10.6084/m9.figshare.12610628.v1. According to the results of T_2_ shown in Figures 3A–D, T_2_ is closely related to the three states of salmon filets at different relaxation times: T_21_ (0.1–1 ms) represents bound water, which is closely related to proteins and other biological macromolecules, T_22_ (43–50 ms) represents the trapped water mainly existing in the tertiary or quaternary structure of the protein or existed between myofibrils and membranes, and T_23_ (464–1,418 ms) represents the free water that has the longest relaxation time and exits outside the myofibrillar fibers (28). The changes of T_21_ were not obvious with the increasing storage time. As the storage time increases, the signal intensity of T_22_ decreased significantly, especially in the T3 group after 6 days of storage. On the other hand, the signal intensity of T_23_ increased rapidly, especially after 6 days of storage. This phenomenon indicated that the trapped water altered to free water during storage, due to the destruction of myofibril structure (29). The changes of T_22_ and T_23_ in the T3 group were more significant than other groups, in accordance with the changes of the TVB-N and TBARS values and bacterial counts.

### Proton MRI

MRI was used to visualize the spatial distribution of moisture in salmon samples under four different storage conditions. In the MRI scan, red (bright) corresponds to regions of high water/proton density, and blue (dark) corresponds to regions of low water/proton density. Fresh salmon filets had the strongest internal signals and the highest moisture content, mainly red and bright yellow. As shown in Figure 3E, with the increasing storage time, the MRI scans of the four groups were gradually changed from bright yellow to blue, and the colors of the T2 and T3 groups were significantly bluer than the other two groups, indicating the decreased of moisture content, corresponding to the previously mentioned changes of T_22_. Temperature fluctuations may promote the migration of water. More water migrates to the outside of the myofibrils, promoting the degradation and denaturation of proteins, resulting in the quality of salmon to decline faster.

### Fatty Acid Profile

The composition and content of fatty acids in salmon are shown in Table 1. A total of 30 fatty acids were detected in salmon filets, including 11 saturated fatty acids (SFAs), eight monounsaturated fatty acids (MUFAs), and 11 PUFAs. The content of unsaturated fatty acids (UFAs) in salmon accounted for about 79% of the total fatty acids. Generally, the higher the content of UFAs, the higher the nutritional value of the food is (6). Among the UFAs in salmon, PUFA accounts for about 32% of the total fatty acids, and MUFA accounts for about 47%. The most abundant fatty acid in salmon was oleic acid (C18:1n9c), followed by linoleic acid (C18:2n6c) and palmitic acid (C16:0).

**Table 1:** The total fatty acids composition and relative contents of salmon filets under four different storage conditions.

**Fatty acid (g/100 g lipids)**	**Storage time (days)**								
	**0 d**	**Con (4 d)**	**Con (8 d)**	**T1 (4 d)**	**T1 (8 d)**	**T2 (4 d)**	**T2 (8 d)**	**T3 (4 d)**	**T3 (8 d)**
C12:0	0.1001	0.1084	0.1079	0.1193	0.0887	0.1084	0.0850	0.1152	0.0934
C13:0	0.0149	0.0156	0.0157	0.0166	0.0130	0.0181	0.0119	0.0137	0.0122
C14:0	2.2740	2.4776	2.4339	2.5620	2.0638	2.5555	1.8728	2.2322	1.9701
C14:1	0.0169	0.0312	0.0299	0.0321	0.0248	0.0308	0.0243	0.0283	0.0248
C15:0	0.2054	0.2417	0.2262	0.2381	0.1822	0.2392	0.1712	0.2088	0.1735
C16:0	12.3576	13.7579	13.5406	14.0297	11.4503	14.2928	10.7844	12.3254	10.7667
C16:1	3.0404	3.4349	3.2523	3.2391	2.8445	3.3210	2.4298	3.0094	2.6674
C17:0	0.3845	0.1947	0.1271	0.1593	0.0985	0.1789	0.1217	0.0906	0.4001
C17:1	0.1674	0.1260	0.1643	0.1784	0.1011	0.1887	0.1285	0.0760	0.1262
C18:0	4.5077	4.1606	3.4166	5.1688	4.1354	5.3129	3.9444	4.9316	4.1437
C18:1n9t	1.0567	0.9374	1.0206	1.1408	0.8064	1.2961	0.8763	0.8766	0.8664
C18:1n9c	34.5058	38.2825	37.3927	38.7214	32.5276	39.9953	29.5512	35.5687	30.5482
C18:2n6t	0.0479	0.0473	0.0741	0.0517	0.0439	0.0492	0.0266	0.0405	0.0331
C18:2n6c	17.7321	19.5138	19.0450	19.4899	16.5007	20.1636	14.9660	18.4532	15.6196
C20:0	0.4547	0.5338	0.4994	0.5583	0.4839	0.5113	0.3930	0.5333	0.4443
C18:3n6	0.2876	0.3393	0.3342	0.3514	0.3023	0.3494	0.2655	0.3390	0.3133
C20:1	5.2859	6.8235	6.1600	6.3603	6.0048	5.8658	4.9388	6.5716	5.3864
C18:3n3	3.4125	2.6435	3.1178	3.4409	2.0603	4.2061	2.5046	2.3758	2.3114
C21:0	0.0592	0.0670	0.0676	0.0759	0.0535	0.0520	0.0552	0.0645	0.0663
C20:2	1.9982	2.2326	2.0781	2.2625	1.9157	2.3525	1.7495	2.0407	1.7831
C22:0	0.2488	0.2745	0.2950	0.3244	0.2407	0.3228	0.2396	0.2575	0.2445
C20:3n6	0.6268	0.6862	0.6707	0.7027	0.5740	0.7035	0.5706	0.6497	0.5518
C22:1n9	0.3500	0.3948	0.3845	0.4113	0.3131	0.4173	0.3039	0.3734	0.3063
C20:3n3	0.6300	0.6487	0.6324	0.8561	0.5864	0.7823	0.6657	0.6309	0.5581
C20:4n6	0.4087	0.4770	0.4636	0.4895	0.3889	0.5001	0.4254	0.4628	0.3714
C23:0	0.0669	0.1059	0.0889	0.0883	0.0578	0.0781	0.0612	0.0757	0.0521
C22:2	0.2327	0.2560	0.2436	0.2789	0.2174	0.2674	0.2080	0.2390	0.2073
C20:5n3	1.8999	2.0425	1.9975	2.1745	1.9760	2.4936	1.8084	2.1709	1.7642
C24:1	0.2911	0.3606	0.3512	0.4076	0.2884	0.3889	0.2977	0.3259	0.2678
C22:6n3	3.7744	4.1343	4.0585	4.1417	3.2119	4.0720	3.7799	3.6447	3.1348
ΣSFA	20.6738	21.9377	20.8189	23.3407	18.8678	23.6700	17.7404	20.8485	18.3669
ΣMUFA	44.7142	50.3909	48.7555	50.4910	42.9107	51.5039	38.5505	46.8299	40.1935
ΣPUFA	31.0508	33.0212	32.7155	34.2398	27.7775	35.9397	26.9702	31.0472	26.6481
EPA+DHA	5.6743	6.1768	6.0560	6.3162	5.1879	6.5656	5.5883	5.8156	4.8990

During storage, the contents of total fatty acids, ΣSFA, ΣMUFA, and ΣPUFA of samples on the 8th day were all lower than those on the 4th day. The fatty acid content in the control samples was more stable than other groups, whose total fatty acids content decreased only by 2.90% when compared to the results on the 8th day with the 4th day. A more significant decrease in fatty acids was found in T2 and T1 groups. The predominant decreased fatty acids in all groups were oleic acid (C18:1n9c), linoleic acid (C18:2n6c), and palmitic acid (C16:0). Linolenic acid (C18:3n3) decreased by more than 40% in T2 and T1 group (4d VS 8d), but it did not change much in the T3 group. It was indicated that the degradation of fatty acid was correlated with the temperature and frequency of temperature fluctuation. The fatty acid is an important precursor of volatile compounds including aldehydes and ketones, leading to odor formation. Therefore, the VOCs were also detected in this study.

### Determination of VOCs

The formations of VOCs are usually contributed by bacterial metabolisms, enzymatic reactions, or lipid auto-oxidation products (30). A total of 25 VOCs were identified in this study, including eleven alcohols, nine aldehydes, four ketones, and one ester (Table 2).

**Table 2:** Main volatile organic compounds (VOCs) and their relative contents (chromatographic peak area × 10^−6^) in salmon filets under four different storage conditions.

**VOCs**	**Relative concentration (Area** **×** **10**^****−6****^**)**								
	**0 d**	**Con (4 d)**	**Con (8 d)**	**T1 (4 d)**	**T1 (8 d)**	**T2 (4 d)**	**T2 (8 d)**	**T3 (4 d)**	**T3 (8 d)**
**Alcohols**									
1-Penten-3-ol	43.47	45.53	53.45	56.23	36.98	52.16	37.42	91.51	47.19
1-Pentanol	7.26	—	—	—	—	—	—	—	—
2-Penten-1-ol, (*Z*)-	11.24	27.07	16.94	23.13	—	35.52	8.13	38.71	15.02
1-Hexanol	1.55	2.55	8.81	3.36	8.91	4.70	10.78	13.08	3.47
1-Octen-3-ol	23.26	77.91	67.76	37.24	43.25	54.11	27.19	89.97	31.34
2-Octyn-1-ol	20.00	57.35	42.92	27.71	28.36	23.51	16.36	71.71	19.38
2-Hexadecanol	0.86	—	—	0.98	1.80	0.51	—	0.94	—
1-Heptanol	24.34	20.28	13.17	8.39	—	—	—	14.97	—
2-Ethyl-1-hexanol	—	—	13.82	7.07	12.17	—	11.20	—	4.89
3-Methyl-1-butanol	—	—	59.91	—	80.44	—	80.83	—	66.23
Phenylethyl alcohol	—	—	7.16	—	58.72	—	31.57	—	10.36
**Aldehydes**									
Hexanal	140.02	261.46	264.38	276.77	230.33	382.05	190.76	438.40	183.94
Heptanal	70.98	48.27	57.81	34.81	58.68	62.67	27.86	89.82	25.49
Benzaldehyde	12.10	15.97	72.14	31.29	51.24	30.60	42.53	46.79	37.00
Octanal	16.24	33.46	29.57	32.15	25.65	31.79	13.63	32.58	13.25
Nonanal	73.74	95.19	68.75	115.30	70.63	113.40	35.32	110.90	31.67
2,6-Nonadienal, (*E, Z*)-	1.76	—	—	—	—	—	—	—	—
Decanal	5.49	6.02	4.15	8.92	5.38	9.34	5.66	4.83	2.61
Propanal	—	6.46	5.27	25.68	10.11	22.70	7.90	—	—
Benzeneacetaldehyde	—	—	26.17	—	36.48	—	28.23	—	29.56
**Ketones**									
2,3-Pentanedione	7.52	—	6.93	—	8.30	39.53	8.32	—	8.08
2,3-Octanedione	46.68	6.17	30.58	12.11	20.70	16.01	11.20	30.45	12.78
2-Butanone	4.25	—	—	—	—	—	—	—	—
2,3-Butanedione	—	—	—	—	14.52	—	13.84	—	16.81
**Esters**									
Acetic acid isopentyl ester	—	—	—	—	—	—	—	—	2.39

— *Indicates not detected*.

Among the volatile alcohol compounds of salmon filets, 1-penten-3-ol, 2-penten-1-ol, (*Z*)-, 1-hexanol, 1-octen-3-ol, and 2-octyn-1-ol increased during storage. 1-Penten-3-ol and 2-octyn-1-ol were the most abundant compounds in salmon filets, which are related to lipid oxidation (mainly caused by the oxidation of UFAs and arachidonic acid by lipoxygenase (31–33). 1-Octen-3-ol is one of the most important contributors of rancid off-flavors because of its low sensorial threshold value so that a little increment was enough for the contribution of lower sensory scores (34).

The highest content of aldehydes was hexanal in fresh salmon, followed by heptanal and nonanal. Hexanal and nonanal were related to the oxidation of linoleic acid and PUFA (35, 36), which were important contributors to meat flavor. In addition, in the later stage of storage, the content of benzaldehyde also increased significantly, and benzeneacetaldehyde also accumulated in the later stage of storage, which was related to the spoilage of salmon filets.

The ketones in salmon filets were much lower than alcohols and aldehydes. The mainly detected compounds of ketones were 2,3-pentanedione, 2,3-octanedione, 2-butanone, and 2,3-butanedione. 2,3-Octanedione was the predominant ketone in fresh salmon, and its content decreased with the increasing storage time. The content of 2,3-butanedione increased significantly during storage, which might be contributed by the metabolic activity of *Pseudomonas* (37).

**Figure 4:**
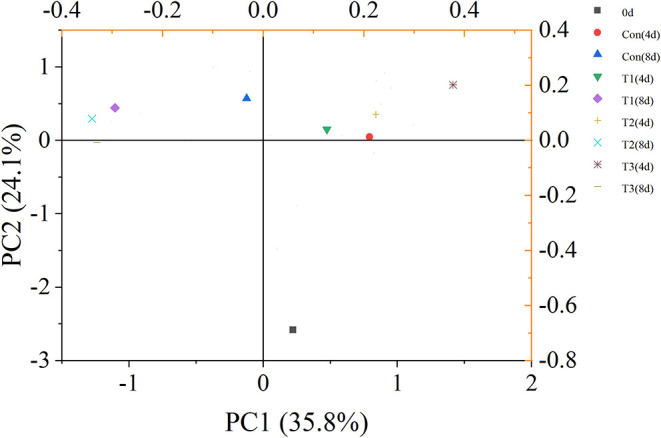
Principal component analysis (PCA) score plot of volatile organic compounds (VOCs) of salmon filets under four different storage conditions.

The principal component analysis showed that VOC variations of different salmon samples were 35.8% [principal coordinates 1 (PC1)] and 24.1% [principal coordinates 2 (PC2)] (Figure 4). The dots of samples were generally distributed in three different quadrants by different storage time. The dot representing fresh salmon filet was far away from other samples. Therefore, the volatile compounds of fresh salmon were significantly different from the samples during storage. The dot of T3 samples on day 4 was also distinguished from other three groups, but the dots of samples became closer to each other after 8 days of storage, indicating that the differences of the content of VOCs between every four groups were much obvious on the 4th day than on the 8th day.

### Relationship Between TBARS Values, Fatty Acid, VOCs, and Water Migration

Table 3 showed the correlation between TBARS values, fatty acids (C16:0, C18:0, C18:1n9c, C18:2n6c, C20:1, ΣSFA, ΣMUFA, ΣPUFA, and EPA + DHA), VOCs (1-penten-3-ol, hexanal, heptanal, nonanal, and 2,3-octanedione), and water migration in salmon filets. The relationship between T_21_ and the TBARS value, fatty acids, and VOCs was quite low, which is closely bound to proteins and has little impact on biological reactions (29). T_22_ was negatively significantly correlated (*p* < 0.05) with octadecanoic acid, 1-penten-3-ol, hexanal, and non-anal. In addition, T_23_ was positively correlated (*p* < 0.05) with 1-penten-3-ol, hexanal, and heptanal. During storage, the trapped water (T_22_) gradually altered to be free water (T_23_) because of the loss of WHC of myofibril protein. The migration of intramyofibrillar water might also lead to the leakage of enzymes such as lipase, lipoxygenase, and protease, which accelerated the degradation and oxidation of fatty acid and proteins and the proliferation of bacteria (38). Therefore, the results indicated that water dynamics had a significant correlation with the fatty acids and VOCs, which could be used to assess and detect the quality changes of salmon.

**Table 3:** Pearson correlation analysis and levels of significance for correlations between TBARS values, fatty acid, and VOCs with T_21_, T_22_, and T_23_.

**Parameter**	**T_**21**_**	**T_**22**_**	**T_**23**_**
TBARS	0.477	0.574	−0.574
C16:0	−0.145	−0.521	0.216
C18:0	−0.062	−0.875^**^	0.343
C18:1n9c	−0.147	−0.576	0.309
C18:2n6c	−0.188	−0.575	0.378
C20:1	−0.074	−0.398	0.648
ΣSFA	−0.189	−0.691^*^	0.279
ΣMUFA	−0.135	−0.567	0.348
ΣPUFA	−0.172	−0.612	0.250
EPA+DHA	−0.022	−0.592	0.251
1-Penten-3-ol	0.079	−0.689^*^	0.837^**^
Hexanal	0.381	−0.807^**^	0.673^*^
Heptanal	−0.224	−0.337	0.703^*^
Nonanal	−0.041	−0.794^*^	0.595
2,3-Octanedione	−0.503	0.071	0.225

Levels of significance are defined as

* p < 0.05 and

** *p < 0.01*.

## Conclusions

This study showed that the sensory score, redness value, and WHC of salmon filets under temperature fluctuation decreased faster than that of filets stored at constant temperature (0 and 4°C), whereas the MBC, PBC, H_2_S-producing bacteria count, TVB-N, and TBARS values gradually increased. LF-NMR ^1^H also showed that water migration was more significant because of temperature shifts. Meanwhile, the fatty acid degraded, and some VOCs including 1-penten-3-ol, hexanal, and 2,3-butanedione contributing to unpleasant rancid flavor increased, which could be a potential indicator of poor quality in salmon. The relationship between T_22_ or T_23_ and other quality indicators had a significant correlation, indicating that LF-NMR ^1^H is a potential non-destructive method to evaluate the quality of seafood. The acceleration of fatty acid degradation and oxidation was observed in samples that suffered frequent temperature fluctuations, and the water migration accelerated the degradation and oxidation of fatty acids. Therefore, the temperature fluctuation during cold chain logistics should be avoided to maintain the nutrients and freshness of salmon.

## Data Availability Statement

The original contributions presented in the study are included in the article/supplementary material, further inquiries can be directed to the corresponding author/s.

## Author Contributions

JX: conceptualization, project administration, and funding acquisition. Y-JY and Y-FQ: methodology. Y-JY: writing—original draft preparation, software, data curation, and visualization. Y-JY, S-PY, and TL: investigation. Y-JY, JX, and CH: validation. JX and Y-FQ: formal analysis, resources, and supervision. Y-FQ: writing—review and editing. All authors contributed to the article and approved the submitted version.

## Conflict of Interest

CH was employed by Nanjing Weigang Dairy Co., Ltd. The remaining authors declare that the research was conducted in the absence of any commercial or financial relationships that could be construed as a potential conflict of interest.
